# Determinants of unmet needs for healthcare and sexual health counselling among Ugandan university students with same-sex sexuality experience

**DOI:** 10.3402/gha.v9.30790

**Published:** 2016-03-30

**Authors:** Markus Larsson, Michael W. Ross, Gilbert Tumwine, Anette Agardh

**Affiliations:** 1 Division for Social Medicine and Global Health, Department of Clinical Sciences, Lund University, Malmö, Sweden; 2 Program in Human Sexuality, Department of Family Medicine, University of Minnesota, Minneapolis, MN, USA; 3 Department of Obstetrics and Gynaecology, St Francis Hospital Nsambya, Kampala, Uganda

**Keywords:** East Africa, sexuality, young people, healthcare access

## Abstract

**Background:**

Research from sub-Saharan Africa has shown that persons with same-sex sexuality experience are at elevated risk for ill health due to sexual risk taking, stigma, and discrimination. However, studies of healthcare seeking among young people in this region with same-sex sexuality experience are limited.

**Objective:**

To identify determinants of unmet healthcare and sexual health counselling needs, respectively, among Ugandan university students with experience of same-sex sexuality.

**Design:**

In 2010, 1,954 Ugandan university students completed a questionnaire assessing socio-demographic factors, mental health, alcohol usage, sexual behaviours, and healthcare seeking. The study population consisted of those 570 who reported ever being in love with, sexually attracted to, sexually fantasised about, or sexually engaged with someone of the same sex.

**Results:**

Findings showed that 56% and 30% reported unmet healthcare and sexual health counselling needs, respectively. Unmet healthcare needs were associated with poor mental health and exposure to sexual coercion (OR 3.9, 95% confidence intervals [CI]: 2.7–5.7; OR 2.0, 95% CI: 1.3–3.0, respectively). Unmet sexual health counselling needs were significantly associated with poor mental health (OR 3.2, 95% CI: 2.1–4.8), exposure to sexual coercion (OR 2.6, 95% CI: 1.7–3.9), frequent heavy episodic drinking (OR 3.3, 95% CI: 1.9–5.8), and number of sexual partners (OR 1.9, 95% CI: 1.04–3.3). The associations between poor mental health, sexual coercion, and unmet healthcare needs (AOR 4.2, 95% CI: 2.1–8.5; AOR 2.8, 95% CI: 1.3–5.8) and unmet needs for sexual health counselling (AOR 3.3, 95% CI: 1.6–7.1; AOR 2.7, 95% CI: 1.4–5.4) persisted after adjustment for socio-demographic factors, number of sexual partners, and frequent heavy episodic drinking.

**Conclusions:**

These findings indicate that exposure to sexual coercion and poor mental health may influence healthcare seeking behaviours of same-sex sexuality experienced students. Targeted interventions that integrate mental health and trauma response are critical to meet the health needs of this population.

## Introduction

Persons in sub-Saharan Africa with experience of same-sex sexuality are at substantial risk for stigma and discrimination (1–3). Discriminatory practices towards individuals with experience of same-sex sexuality such as blackmailing, threats, and denial of services by healthcare workers have been reported across the continent (4, 5). Due to adverse experiences and/or negative expectations concerning healthcare, people with experience of same-sex sexuality may delay treatment seeking and avoid healthcare services, thereby putting themselves at increased risk for ill health (6). In a study of 537 men who have sex with men (MSM) in Malawi, Botswana, and Namibia, 18.5% revealed that they were afraid of seeking healthcare services due to fear of stigma and repercussions (7). Homophobic stigma has been identified as a critical obstacle for achieving the universally agreed zeros related to HIV and AIDS, that is, zero new infections, zero new AIDS-deaths, and zero discrimination (8). Thus, in sub-Saharan Africa in particular, persons with experience of same-sex sexuality may have unmet healthcare needs.

Little is known about the unmet healthcare needs of persons with experience of same-sex sexuality in Uganda. Uganda has a long history of negative sanctions towards same-sex activity. The country has during the last decade witnessed a dramatic development whereby a law with harsher sanctions, the Anti-Homosexuality Bill, was ratified in 2014 and retracted only a few months later on technical grounds (9). A proposal of a new law is under discussion, but the status of this proposal currently remains unclear (10). It is likely that these developments have reduced access to healthcare for Ugandan persons with same-sex sexuality experience. Consequently, unmet healthcare needs may be especially high in this population. Research that has focused exclusively on MSM in Uganda has revealed that this population has high HIV and STI prevalence (12.9% and 13.5%, respectively), that alcohol use before sex is common, and that there is a risk of discrimination and rejection of healthcare by service providers (11–13). Nevertheless, increased HIV is primarily a health concern of men with same-sex activity, and less is known about health needs of women with same-sex activity.

Youth is a period of sexual exploration, and same-sex attraction may be common in young adults and in student populations (14–16). Although previous reports suggest that students at the Mbarara University of Science and Technology (MUST) generally have high rates of unmet healthcare needs and unmet sexual health counselling needs (17, 18), those with experience of same-sex sexuality had elevated risk for poor health outcomes and unmet healthcare needs compared to the general student population (19). The findings showed by Agardh et al. indicating increased risk for poor mental health, substance abuse, risky sexual behaviour, exposure to sexual coercion, and unmet sexual health counselling needs among students with same-sex sexuality are thus particularly alarming (19). However, research concerning healthcare access among young adults with experience of same-sex sexuality in sub-Saharan Africa is scarce. In order to be able to meet the challenge of adequate healthcare provision for persons with same-sex sexuality, it is important to identify factors that are associated with unmet healthcare needs in this population.

The aim of the current study was to identify background and lifestyle characteristics associated with unmet healthcare needs and unmet needs for sexual health counselling among Ugandan university students with experience of same-sex sexuality. Unmet healthcare needs and unmet needs for sexual health counselling were examined in relationship to the following characteristics: alcohol consumption, sexual behaviour, age of sexual debut, condom use, history of sexual coercion, and mental health status. It was considered important to study unmet healthcare needs and unmet needs for sexual health counselling as separate measures, as individuals may have different concerns with regard to their general health and their sexuality and sexual lives. Findings from the study can be used to inform interventions that target determinants of health at both structural and individual levels in this population.

## Method

### Population and setting

#### Study participants

Study participants were recruited from MUST, a public university located in southwest Uganda, in 2010. Mbarara is the administrative capital city of Mbarara district and had a population of 69,000 in 2002, according to the last census preceding the time of the study (20). MUST is one of the larger universities in the country, established in 1989 (21). All undergraduate students (*n*=2,706) from the four faculties of medicine, science, development studies, and computer science were invited to participate in the study. Ethical approval for the study was granted by the Institutional Review Committee at MUST.

### Procedures

#### Survey instrument

Students who volunteered to participate in the survey signed a consent form and completed a 132-item questionnaire, distributed in lecture halls at MUST by the research team. The distribution of the questionnaire was conducted by the research team, and no faculty members participated. Prior to study start, the questionnaire had been pre-tested both individually and in focus groups. The questions regarding substance use, mental health, and sexuality had previously been validated (22–25). The language used was English, which is the teaching language at MUST. Contact details for the principal investigator and a research assistant were provided in case any personal concerns would arise after completing the questionnaire. When returning the questionnaire, students separated the consent page from the questionnaire and placed them in different boxes to ensure anonymity. A total of 1,954 students completed the questionnaires, representing 72% of the undergraduate students at the university. The sample in the current study consisted of the subset of individuals, who had reported experience of same-sex sexuality (570 students).

### Measures

#### Independent measures

The questionnaire included four questions that assessed emotional and behavioural experiences representing same-sex sexuality. This strategy, whereby components of same-sex sexuality were deconstructed into simplified types of experiences to avoid connoted words and terms, has previously been employed by Lewin et al. in their study of sexuality in Sweden (25). The questions were: ‘If you think of the people you have been in love with, what gender were they?’, ‘If you think of the people you have been sexually attracted to, what gender were they?’, ‘If you think of the people you have sexually fantasised about, what gender were they?’, and ‘If you think of the people you have had sexual relations with, what gender were they?’. The options were recorded as ‘always female’, ‘usually female but sometimes male’, ‘male/female equally’, ‘usually male but sometimes female’, and ‘always male’, and then dichotomised into ‘only heterosexual experience (i.e. solely with the opposite gender)’ and ‘mixed experience’.

For the purpose of multivariable analysis, an aggregated variable was created, that is, ‘experience of same-sex sexuality’. This variable included those who responded ‘yes’ to ever been in love with, or ever been sexually attracted to, or ever sexually fantasised about, or ever had sexual relations with someone of the same gender.

*Age:* Age was dichotomised as two age groups: ‘≥24 years’ and ‘<24 years’. This division is consistent with the United Nation's definition of youth, that is, those aged 15–24 (26).

*Area of origin*: This was classified as ‘rural’ and ‘urban/peri-urban’.

*Educational level of head of household*: This was dichotomised as follows: ‘≤primary school’ including those who had completed primary school as well as those whose educational level was lower, and ‘>primary school’ for those who had any kind of education higher than primary school.

*Frequent heavy episodic drinking* was based on the question, ‘How often do you drink six “glasses” or more on the same occasion?’ The options were recorded as ‘daily or almost daily’, ‘every week’, ‘every month’, ‘more seldom than once a month’, and ‘never’. This variable was then dichotomised by combining the first three alternatives as ‘yes’ (risk for frequent heavy episodic drinking) and the last two as ‘no’ (no risk for frequent heavy episodic drinking).

*Mental health status*: The Hopkins Symptom Checklist-25 (HSCL-25) was used to measure psychological distress (27). This screening instrument contains 10 questions concerning symptoms of anxiety and 15 questions concerning symptoms of depression. Ten items from the Symptom Checklist-90 (SCL-90), a self-reported symptom inventory of psychopathology, were further included to assess signs of psychoticism (28). Both checklists have previously been used in sub-Saharan Africa to measure mental health (24–29). For each symptom, respondents used a four-point scale (1 for ‘not at all’, 2 for ‘little’, 3 for ‘quite a bit’, and 4 for ‘extremely’) in answer to the question, ‘How much has this problem bothered or distressed you during the last week, counting today?’. To obtain the total mental health mean score for each participant, their total scores for depression, anxiety, and psychoticism were divided by the number of items they responded to. The scores were then dichotomised as ‘high’ versus ‘low’, where ‘high’ represented ‘poor mental health’ and ‘low’ represented ‘satisfactory mental health’, based on the total distribution's median-split.

*Age of sexual debut* was based on the question ‘At what age did you have sexual intercourse for the first time?’ and was dichotomised as ‘≤15 years’ and ‘>15 years’.

*Number of sexual partners during the last 12 months* was based on the question ‘How many sexual partners have you had during the last 12 months?’ and was dichotomised as ‘≤1’ and ‘≥2’.

*Condom use on latest occasion of sexual intercourse* was determined by asking ‘Did you use any method for avoiding sexually transmitted diseases on your latest occasion of sexual intercourse?’, and the response alternatives ‘no’ and ‘yes, other’ were dichotomised as ‘no’ and ‘yes, condom’ as ‘yes’.

*Exposure to sexual coercion* was based on a response of ‘yes’ to any of the following questions: ‘You have been *forced* to show your sexual organ’, ‘Someone has *forced* you to let them touch your sexual organ’, ‘Someone *forced* you to let them suck or lick your sexual organ’, ‘Someone has *forced* you to let them show you their own sexual organ’, ‘You have been *forced* to watch someone masturbate’, ‘You have been *forced* to masturbate someone’, ‘You have been *forced* to take part in oral sex or to lick someone's sexual organ’, ‘You have been *forced* to take part in sexual intercourse with the penis in the vagina, or someone has inserted an object into your vagina’, or ‘You have been *forced* to pose for a sex photo or sex film’. This assessment method has previously been used in both Uganda and Sweden (25–30). Respondents who reported yes to one or to several of these questions were dichotomised as ‘yes’ (exposure to sexual coercion), and the rest were dichotomised as ‘no’ (no exposure to sexual coercion).

#### Dependent measures (healthcare needs)

The main outcome variables were unmet healthcare needs and unmet needs for sexual health counselling, respectively. These were assessed through the following questions: ‘Have you at any time during the last three months experienced a health problem but abstained from seeking care?’ and ‘Have you at any time during the last three months experienced a sexual health problem but abstained from seeking health care?’. The response alternatives ‘yes, several times’ and ‘yes’ were dichotomised as ‘yes’ and ‘no’ as ‘no’.

### Statistical analysis

Statistical analysis was conducted using Stata Version 12 (StataCorp. College Station, TX, USA). The statistical analysis was carried out in three stages. First, frequencies and significance values (*p* values) were calculated for the categorical variables, disaggregated by gender. As a second step, simple logistic regression analyses, stratified for male and female students, were used to obtain crude odds ratios (OR) and 95% confidence intervals (CI) for the associations between socio-demographic factors, mental health, frequent heavy episodic drinking, sexual behaviours, and exposure to sexual coercion (independent variables) and the two outcome variables represented by unmet healthcare and unmet sexual health counselling needs, respectively. Significance levels were set at 5% with CIs.

Multiple logistic regression analyses, adjusted for age, area of origin, and household head's level of education, were performed in the total sample and stratified by gender as a last step to calculate the adjusted odds ratios (AOR) and 95% CI for the association between mental health, frequent heavy episodic drinking, number of sexual partners, and exposure to sexual coercion, and the two outcome variables represented by unmet healthcare needs and unmet needs for sexual health counselling. Missing values represent incomplete answers for at least one of the variables included in the model and were excluded from the analysis.

## Results

Among those who responded to the questionnaire, 570 persons answered ‘yes’ to at least one of the questions pertaining to same-sex sexuality; 54% were females (*n*=305) and 46% were males (*n*=265). Thus, 499 (232 males; 267 females) reported ever being in love with someone of the same gender, 182 (72 males, 110 females) that they ever been sexually attracted to someone of the same gender, 171 (74 males, 97 females) that they ever had sexually fantasised about someone of the same gender, and 111 (males 52; females 59) that they ever had had sexual relations with someone of the same gender.

Table 1 shows the frequency distributions for the background and lifestyle characteristics that were hypothesised to influence the health needs among students with experience of same-sex sexuality. As indicated in this table, reports of frequent heavy episodic drinking and two or more sexual partners during the last 12 months were more common among male students. A larger percentage of females (40.3%) than males (34.4%) reported exposure to sexual coercion. More than half (56%) of the study population reported experience of unmet healthcare needs during the last 3 months and about one-third (30.3%) reported unmet needs for sexual health counselling during the last 3 months.

**Table 1:** Distribution of background and lifestyle characteristics among a sample of Ugandan university students with same-sex sexuality experience

Variables	All (*n*=570) *n* (%)	Males (*n*=265) *n* (%)	Females (*n*=305) *n* (%)	Chi-square *p*-value
Age				
<24 years	399 (72.8)	168 (65.9)	231 (78.8)	<0.001
≥24 years	149 (27.2)	87 (34.1)	62 (21.2)	
Area of growing up				
Urban	248 (43.7)	104 (39.5)	144 (47.2)	0.066
Rural	320 (56.3)	159 (60.5)	161 (52.8)	
Educational level of parent				
>Primary school	383 (67.9)	158 (60.1)	225 (74.8)	<0.001
≤Primary school	181 (32.1)	105 (39.9)	76 (25.2)	
Mental health				
Satisfactory	235 (43.7)	120 (48.4)	115 (39.7)	0.042
Poor	303 (56.3)	128 (51.6)	175 (60.3)	
Missing	32	17	15	
Frequent heavy episodic drinking				
No	450 (88.2)	199 (81.9)	251 (94)	<0.001
Yes	60 (11.8)	44 (18.1)	16 (6)	
Missing	60	22	38	
Age of sexual debut^a^				
≥15 years	242 (86.1)	116 (83.5)	126 (88.7)	0.201
<15 years	39 (13.9)	23 (16.5)	16 (11.3)	
Missing	51	16	35	
Number of sexual partners in the last 12 months^a^				
≤1	158 (65.3)	53 (48.2)	105 (79.5)	<0.001
≥2	84 (34.7)	57 (51.8)	27 (20.5)	
Missing	90	45	45	
Condom use during the last sexual intercourse^a^				
Yes	187 (64.7)	101 (73.2)	86 (57)	0.004
No	102 (35.3)	37 (26.8)	65 (43)	
Missing	43	17	26	
Exposed to sexual coercion				
No	306 (62.5)	149 (65.6)	157 (59.7)	0.176
Yes	184 (37.5)	78 (34.4)	106 (40.3)	
Missing	80	38	42	
Had health problem but abstained from healthcare for the last 3 months				
No	228 (44)	110 (46.4)	118 (42)	0.313
Yes	290 (56)	127 (53.6)	163 (58)	
Missing	52	28	24	
Had sexual health problem but abstained from healthcare for the last 3 months				
No	355 (69.7)	167 (70.2)	188 (69.4)	0.845
Yes	154 (30.3)	71 (29.8)	83 (30.6)	
Missing	61	27	34	

a Only analysed among individuals who ever had had sexual intercourse.

Table 2 shows the results of the simple logistic regression analyses of the associations between socio-demographic factors, poor mental health, frequent heavy episodic drinking, sexual behaviours, and exposure to sexual coercion in relation to unmet needs for healthcare and unmet needs for sexual health counselling services during the last 3 months, for the total sample and stratified by gender. Poor mental health was significantly associated with unmet healthcare needs (OR 3.9, 95% CI: 2.7–5.7) and unmet sexual health counselling needs (OR 3.2, 95% CI: 2.1–4.8) in the total sample, and also among both male and female students separately. Students with frequent heavy episodic drinking had a significantly higher risk for both unmet healthcare needs (OR 1.9, 95% CI: 1.02 – 3.5) and unmet sexual health counselling needs (OR 3.3, 95% CI: 1.9–5.8). Furthermore, having more than one sexual partner during the last 12 months was significantly associated with unmet healthcare needs among male students (OR 2.7, 95% CI: 1.2–6.3) but not female students and with unmet sexual health counselling needs among all students (OR 1.9, 95% CI: 1.04–3.3). Students exposed to sexual coercion were at higher risk for unmet healthcare needs (OR 2.0, 95% CI: 1.3–3.0) and unmet sexual health counselling needs (OR 2.6, 95% CI: 1.7 – 3.9) in the total sample, and also among male and female students separately. Furthermore, no associations were found between age of sexual debut or condom use at last sexual intercourse and unmet healthcare needs, unmet sexual health counselling needs, respectively.

**Table 2:** Associations (crude odds ratios, 95% confidence intervals) between socio-demographic factors, frequent heavy episodic drinking, mental health, sexual behaviour and exposure to sexual coercion, and ‘unmet healthcare needs’ and ‘unmet sexual health counselling needs’

	Unmet healthcare needs All (*N*=570)	Males (*n*=265)	Females (*n*=305)	Unmet sexual health counselling needs All (*N*=570)	Males (*n*=265)	Females (*n*=305)
	
Variable	Odds ratios (95% CI)	Odds ratios (95% CI)	Odds ratios (95% CI)	Odds ratios (95% CI)	Odds ratios (95% CI)	Odds ratios (95% CI)
Age						
<24 years	1 (ref.)	1 (ref.)	1 (ref.)	1 (ref.)	1 (ref.)	1 (ref.)
≥24 years	0.8 (0.5–1.2)	1.0 (0.6–1.7)	0.7 (0.4–1.2)	1.1 (0.7–1.7)	1.4 (0.8–2.6)	0.8 (0.4–1.6)
Area of growing up						
Urban	1 (ref.)	1 (ref.)	1 (ref.)	1 (ref.)	1 (ref.)	1 (ref.)
Rural	1.1 (0.8–1.6)	1.1 (0.7–1.9)	1.1 (0.7–1.8)	1.3 (0.9–1.9)	1.4 (0.8–2.5)	1.2 (0.7–2.0)
Educational level of parent						
>Primary school	1 (ref.)	1 (ref.)	1 (ref.)	1 (ref.)	1 (ref.)	1 (ref.)
≤Primary school	1.4 (0.9–2.0)	**1.8 (1.1–3.0)**	1.1 (0.6–1.9)	0.9 (0.6–1.4)	0.9 (0.5–1.6)	0.9 (0.5–1.6)
Mental health						
Satisfactory	1 (ref.)	1 (ref.)	1 (ref.)	1 (ref.)	1 (ref.)	1 (ref.)
Poor	**3.9 (2.7–5.7)**	**4.7 (2.7–8.1)**	**3.3 (2.0–5.5)**	**3.2 (2.1–4.8)**	**4.1 (2.2–7.7)**	**2.5 (1.4–4.5)**
Frequent heavy episodic drinking						
No	1 (ref.)	1 (ref.)	1 (ref.)	1 (ref.)	1 (ref.)	1 (ref.)
Yes	**1.9 (1.02–3.5)**	2.1 (0.9–4.4)	1.9 (0.6–6.3)	**3.3 (1.9–5.8)**	**3.6 (1.8–7.2)**	**3.2 (1.1–8.9)**
Age of sexual debut^a^						
≥15 years	1 (ref.)	1 (ref.)	1 (ref.)	1 (ref.)	1 (ref.)	1 (ref.)
<15 years	1.5 (0.7–3.2)	2.3 (0.8–6.9)	1.0 (0.3–2.8)	1.1 (0.5–2.3)	1.4 (0.5–3.7)	0.8 (0.3–2.6)
Number of sexual partners in last 12 months^a^						
≤1	1 (ref.)	1 (ref.)	1 (ref.)	1 (ref.)	1 (ref.)	1 (ref.)
≥2	1.7 (0.9–3.0)	**2.7 (1.2–6.3)**	1.0 (0.4–2.4)	**1.9 (1.04–3.3)**	2.0 (0.9–4.7)	2.1 (0.8–5.2)
Condom use during last sexual intercourse^a^						
Yes	1 (ref.)	1 (ref.)	1 (ref.)	1 (ref.)	1 (ref.)	1 (ref.)
No	1.2 (0.7–1.9)	2.0 (0.9–4.7)	0.8 (0.4–1.6)	1.3 (0.8–2.2)	1.1 (0.5–2.6)	1.5 (0.7–3.0)
Exposure to sexual coercion						
No	1 (ref.)	1 (ref.)	1 (ref.)	1 (ref.)	1 (ref.)	1 (ref.)
Yes	**2.0 (1.3–3.0)**	**2.3 (1.2–4.2)**	**1.8 (1.1–3.1)**	**2.6 (1.7–3.9)**	**2.9 (1.6–5.3)**	**2.3 (1.3–4.1)**

a Only analysed among individuals who ever had had sexual intercourse.

Values presented in bold were significant at least at the 5% level.

Factors shown to be significantly related to unmet healthcare needs and unmet needs for sexual health counselling were entered into a multiple logistic regression analysis, adjusted for age, area of origin and household head's level of education and stratified by gender. Table 3 presents the OR obtained in the fully adjusted model for the associations between factors, that is, mental health, frequent heavy episodic drinking, number of sexual partners during the last 12 months, and exposure to sexual coercion, and unmet healthcare needs and unmet needs for sexual health counselling. The significant associations between poor mental health and unmet healthcare and unmet sexual health counselling needs persisted in the fully adjusted model, both in the total sample (AOR 4.2, 95% CI: 2.1–8.5 and AOR 3.3, 95% CI: 1.6–7.1, respectively) and among males and females separately. Exposure to sexual coercion remained significantly associated with unmet healthcare needs among females (AOR 3.5, 95% CI: 1.3–9.7) but not among males and remained significantly associated with sexual health counselling needs among both male and female students (males: AOR 3.2, 95% CI: 1.1–9.6, females: AOR 2.6, 95% CI: 1.01–6.5).

**Table 3:** Associations (adjusted odds ratios, 95% confidence intervals) between frequent heavy episodic drinking, mental health, number of sexual partners, exposure to sexual coercion, and ‘unmet healthcare needs’ and ‘unmet sexual health counselling needs’ among a sample of Ugandan university students with same-sex sexuality experience

	Unmet healthcare needs All (*N*=570)	Males (*n*=265)	Females (*n*=305)	Unmet sexual health counselling needs All (*N*=570)	Males (*n*=265)	Females (*n*=305)
	
Variable	Adjusted odds ratios (95% CI)	Adjusted odds ratios (95% CI)	Adjusted odds ratios (95% CI)	Adjusted odds ratios (95% CI)	Adjusted odds ratios (95% CI)	Adjusted odds ratios (95% CI)
Mental health						
Satisfactory	1 (ref.)	1 (ref.)	1 (ref.)	1 (ref.)	1 (ref.)	1 (ref.)
Poor	**4.2 (2.1–8.5)**	**5.2 (1.8–15.3)**	**4.0 (1.5–10.5)**	**3.3 (1.6–7.1)**	**5.2 (1.5–18.7)**	**3.0 (1.1–8.2)**
Frequent heavy episodic drinking						
No	1 (ref.)	1 (ref.)	1 (ref.)	1 (ref.)	1 (ref.)	1 (ref.)
Yes	2.9 (0.9–9.0)	2.3 (0.6–8.9)	4.3 (0.4–46.6)	1.3 (0.5–3.0)	1.8 (0.6–5.5)	0.8 (0.1–4.1)
Sexual partners in last 12 months						
≤1	1 (ref.)	1 (ref.)	1 (ref.)	1 (ref.)	1 (ref.)	1 (ref.)
≥2	1.3 (0.6–2.9)	2.9 (0.9–9.1)	0.5 (0.2–1.9)	1.4 (0.7–2.9)	1.4 (0.5–4.2)	2.0 (0.6–6.4)
Exposure to sexual coercion						
No	1 (ref.)	1 (ref.)	1 (ref.)	1 (ref.)	1 (ref.)	1 (ref.)
Yes	**2.8 (1.3–5.8)**	1.9 (0.6–5.8)	**3.5 (1.3–9.7)**	**2.7 (1.4–5.4)**	**3.2 (1.1–9.6)**	**2.6 (1.01–6.5)**

Adjusted for age, area of origin, and household head's level of education.

All variables were mutually adjusted for one another.

Values presented in bold were significant at least at the 5% level.

However, the associations between frequent heavy episodic drinking, two or more sexual partners during the last 12 months and unmet healthcare, unmet sexual health counselling needs, respectively, were no longer significant when adjusted for all potential confounders.

## Discussion

This is the first study, to our knowledge, to investigate the unmet healthcare and sexual health counselling needs among young adults with experience of same-sex sexuality in sub-Saharan Africa. Findings reveal a high proportion of unmet needs in this group, whereby one in two and one in three reported unmet healthcare needs and unmet sexual health counselling needs, respectively, during the last 3 months. In the fully adjusted model, poor mental health and exposure to sexual coercion were significantly associated with reported unmet healthcare and unmet sexual health counselling needs. Unmet healthcare needs have been found to contribute to health disparities in this population (11, 12) and challenge Uganda's efforts to improve the health of key populations.

The proportion of students with unmet healthcare (56%) and sexual health counselling needs (30%) in our study was higher than in a previous study of the general student population at the same university (18). Boltena et al. reported in their study that 41% of the students had unmet healthcare needs and 21% had unmet sexual health counselling needs (18). The major obstacle identified was ‘acceptability’, that is, whether the students believed they could receive help for the problem and whether they knew of, and had confidence in, existing service providers (18). Previous research has revealed that in sub-Saharan Africa, persons with experience of same-sex sexuality lack trust and confidence in healthcare providers not only due to perceived lack of competency among the providers, but also due to experience of maltreatment (3–5, 31, 32). Whether the participants in the current study refrained from seeking healthcare due to such experiences or other factors cannot be ascertained on the basis of the data available. However, reluctance to seek care for health problems might indirectly suggest that persons with same-sex sexuality perceive existing healthcare services as non-optimal or not suited to their specific needs. This implies that there is a potential need for tailored health services to help address the challenges this population faces. However, tailoring for same-sex sexuality issues in Uganda requires careful consideration to avoid stigmatisation and potential closedown of services, as was the case for one clinic that was raided by authorities in 2014 due to accusations of recruiting homosexuals (33).

Sexual health counselling services are by their very nature concerned with private matters/sensitive issues and require a high degree of trust between the patient and the provider. During the time of the data collection, a heated public debate against homosexuality followed the submission of the Anti-Homosexual Bill to the Ugandan parliament in 2009 (34). Tabloid press published identities, photographs, and addresses of perceived lesbians and gays, and demonstrations in favour of the Anti-Homosexual Bill took place (35). Such incidents inevitably contribute to a climate of fear of repercussions if exposed as homosexual by a healthcare worker, and such fears could affect healthcare-seeking negatively. Perceived or felt stigma (36), the fear of becoming stigmatised, has previously been found to be associated with fear of seeking healthcare (61.7%, 95% CI=54.0–69.0%) in a sample of 323 MSM in Swaziland (37). In Uganda also, stigma in healthcare has been identified as a barrier for MSM to seek healthcare services (11). This may indicate that students with experience of same-sex sexuality in our sample refrained from sexual health counselling services out of fear of being exposed as ‘gay’ or ‘lesbian’.

Our findings revealed that poor mental health and exposure to sexual coercion were independently associated with unmet healthcare and sexual health counselling needs. These findings are similar to those of Kayagaba et al. in their study of the general student population at the MUST (17). Students with poor mental health and exposure to sexual coercion were at higher risk of reporting unmet needs for sexual health counselling services compared to those without such previous experiences. Studies among the US college students report that victims of sexual coercion tend to delay or avoid reporting the crime due to feelings of guilt, shame, and fear of not being taken seriously by the healthcare provider (38, 39). In addition, studies have shown that the strong stigmatisation of male sexual assault prevents victims from seeking assistance (40, 41). Based on our findings, we cannot determine when the abuse occurred or the identity of the perpetrator. However, the present study raises the possibility that the unfavourable social discourse around homosexuality in Uganda in 2010 (34) might have dissuaded victims from seeking healthcare due to the fear of having their own sexuality questioned. Although we have no explanation for the difference observed concerning unmet healthcare needs in males with experience of same-sex sexuality, it is likely that contextual factors played a role.

A large body of literature has explored the relationship between stress, depression, victimisation, and homophobic stigma among persons with experience of same-sex sexuality (42–45). Stressors that are unique for this population, induced by a hostile and homophobic culture, could have a negative influence on their mental health (46, 47). Internalised homonegativity (IH) is the process whereby a person with same-sex sexuality develops self-stigma towards his or her own sexuality as result of the prevailing prejudices, attitudes, and practices around same-sex sexuality (48). Previous research has found a correlation between higher levels of IH and avoidance of HIV-testing services among MSM (49, 50). This might imply that IH could also affect healthcare seeking in general for this population. Studies from African universities have found the campus climate towards same-sex sexuality to be conservative and hostile, with an institutionalised tolerance for homonegativity (51–53). Although institutionalised and IH may also have influenced the current findings, little is known about the actual prevalence of negative attitudes towards same-sex sexuality at the university where the current data were collected.

Findings from this study reveal that there is an unmet need for healthcare and sexual health counselling services among a particularly vulnerable group of same-sex sexuality experienced youth, that is, those with poor mental health or with previous exposure to sexual coercion. Future healthcare interventions must include sensitised personnel, trained in the specific needs of young adults with experience of same-sex sexuality, including how to identify cases of sexual coercion and how to counsel victims. Promising results from Kenya have shown that the Internet could be an appropriate platform for healthcare providers to acquire improved knowledge about the sexual health of MSM (54). Further assessment is required to explore whether such technology-led initiatives could be extended to include the needs of the current study population. Finally, to ensure that research tools are tailored to the reality, there is a need to consider the variety of experiences that reflect same-sex sexuality in order to design better survey instruments with appropriate items and response alternatives. Additional research is also required that specifically examines reasons for not seeking healthcare in this population.

### Limitations of the study

This is the first study to examine determinants of unmet healthcare and sexual health counselling needs among individuals with same-sex sexuality conducted in Uganda, utilising information from a comprehensive survey with high participation rates with both males and females. The study has, however, several limitations, and thus, the results should be interpreted with caution. An important limitation was the cross-sectional nature of the study design. Although certain factors were found to be associated with unmet health needs in the study sample, no claims of causal inference could be made. Secondly, the sample population (i.e. university students) was not a national probability sample, which limits the external generalisability of the findings. In 2010, only 5.4% of Uganda's population was enrolled in a tertiary institution (55). Yet, during the period 2006–2010, student enrolment increased by 34% (55). University students are thus a growing population in Uganda and as such, our findings contribute to an understanding of healthcare-seeking among same-sex sexuality individuals attending higher education. Thirdly, some of the CIs obtained when the analyses were stratified by gender were very broad, due to small sample size. Moreover, the university-wide survey from which the current data were derived did not intentionally target students with experiences of same-sex sexuality but rather sought to examine the prevalence of a variety of sexual behaviours in a general student population. In this regard, the definition of age at sexual debut, number of sexual partners, and condom use might have been too narrow as these variables were based on sexual intercourse *per se* and may not have reflected other types of sexual activity. Therefore, same-sex sexuality might have been underreported, thus possibly affecting the associations with unmet healthcare needs. Future studies of same-sex sexuality will need to ask more detailed questions about risk perceptions, attitudes, and sexual practices among this population to better understand the extent and nature of these. Same-sex sexuality may also have been underreported due to social desirability bias, especially in light of the heightened debate following the Anti-Homosexuality Bill in 2009 (34). Nevertheless, an attempt to mitigate potential underreporting was made by avoiding words in the questionnaire that could have negative associations.

## Conclusions

This study identified poor mental health and previous exposure to sexual coercion as prominent factors for not seeking healthcare and sexual health counselling, respectively, among Ugandan university students with experiences of same-sex sexuality. Thus, targeting mental ill health and sexual coercion could be of critical importance for improving the health of persons with same-sex sexuality. Interventions that develop healthcare providers’ understanding of same-sex sexuality and their counselling skills could have important positive effects on the health of the study population as well as the general youth population.

## Authors' contributions

ML drafted the manuscript, analysed and interpreted the data, and was involved in the conceptualisation and design of the study. AA was involved in the conceptualisation of the study; developed the survey instrument; collected the data; and contributed to the study design, data analysis, data interpretation, and manuscript preparation. MR contributed to the interpretation and analysis of the data. GT contributed to the survey instrument and participated in data collection and data interpretation. All authors contributed to the writing and approved the final version of the manuscript.
